# Increasing teacher motivation and supervision is an important but not sufficient strategy for improving praziquantel uptake in *Schistosoma mansoni* control programs: serial cross sectional surveys in Uganda

**DOI:** 10.1186/1471-2334-13-590

**Published:** 2013-12-13

**Authors:** Simon Muhumuza, Anne Katahoire, Fred Nuwaha, Annette Olsen

**Affiliations:** 1 School of Medicine, Child Health and Development Center, Makerere University, Kampala, Uganda; 2 School of Public Health, Makerere University, Kampala, Uganda; 3 Faculty of Health and Medical Sciences, Section for Parasitology and Aquatic Diseases, University of Copenhagen, Copenhagen, Denmark

**Keywords:** Schistosomiasis, Uptake of praziquantel, School children, Uganda

## Abstract

**Background:**

Realization of the public health benefits of mass drug administration (MDA) for the control of schistosomiasis depends on achieving and maintaining high annual treatment coverage. In Uganda, the uptake of preventive treatment for schistosomiasis among school-age children in 2011 was only 28%. Strategies are needed to increase uptake.

**Methods:**

Serial cross-sectional surveys were conducted at baseline (after MDA in 2011) and at follow-up MDA in 2012 where teacher motivation was provided and supervision strengthened in Jinja district of Uganda. Uptake of praziquantel was assessed in 1,010 randomly selected children from 12 primary schools during the baseline survey and in another set of 1,020 randomly selected children from the same primary schools during the follow-up survey.

**Results:**

Self-reported uptake of praziquantel increased from 28.2% (95% CI 25.4%-30.9%) at baseline to 48.9% (95% CI 45.8%-52.0%) (p < 0.001) at follow-up. Prevalence and intensity of *Schistosoma mansoni* infection were unchanged and moderate on both occasions; 35.0% (95% CI: 25.4%-37.9%) and 32.6% (95% CI: 29.6%-35.5%) (p = 0.25) and 156.7 eggs per gram of stool (epg) (95% CI: 116.9-196.5) and 133.1 epg (95% CI: 99.0-167.2) (p = 0.38), respectively. There was no change in the proportion of children reporting side effects attributable to praziquantel at baseline (49.8%, 95% CI 43.8%-55.8%) and at follow-up (46.6%, 95% CI 42%.1-51.2%) (p = 0.50) as well as in the proportion of children with correct knowledge of schistosomiasis transmission and control between the baseline (45.9%, 95% CI 42.7%-73.7%) and follow-up (44.1%, 95% CI 41.0%- 47.2%) (p = 0.42).

**Conclusion:**

Although teacher motivation and supervision to distribute treatment increased the uptake of praziquantel among school-age children, the realized uptake is still lower than is recommended by the World Health Organization (WHO) and apparently too low to affect the prevalence and intensity of schistosomiasis among the children. Additional measures are needed to increase uptake of praziquantel if school-based MDA is to achieve the objective of preventive chemotherapy.

## Background

The World Health Organization (WHO) recommends treatment programs for schistosomiasis to target school-age children who could be reached through the primary school system, in collaboration with the education sector [1]. This method is considered affordable and cost-effective [2,3]. The goal is to provide regular treatment to at least 75% of school-age children at risk of morbidity [4,5]. Successful schistosomiasis control programs have been implemented in many countries [4-9]. Varying levels of success have been reported in many developing countries where regular chemotherapy with praziquantel is implemented as the main strategy for schistosomiasis control [9-13].

In Uganda, the national program for the control of schistosomiasis adopted the WHO recommendations in 2003 and has since implemented school-based MDA with praziquantel in high burden communities [14]. Realization of the public health benefits of MDA, such as reduction in prevalence, intensity of infection and morbidity attributable to schistosomiasis, will depend on achieving and maintaining high annual treatment coverage. However, previous studies in Uganda have reported a very low uptake of praziquantel in some areas [11,15,16]. A baseline of the present study conducted in primary schools of Jinja district showed that less than 30% of school children took praziquantel during the 2011 MDA [11]. Thus, measures are needed to increase uptake of MDA in Uganda. Following the very low uptake in 2011, the Ugandan national program attempted to remedy the situation by adopting new strategies for distribution of praziquantel. This paper reports the effects of the new strategy implemented in 2012 on levels of uptake, children’s knowledge on schistosomiasis prevention and prevalence and intensity of schistosomiasis before and after implementation.

## Methods

### Study design

Serial cross-sectional surveys were carried out in 2011 and 2012 in Jinja district. A survey before the 2011 MDA (hereafter referred to as the baseline) was conducted in September 2011, six months after the 2010 MDA. Another survey was conducted in July 2012, three weeks after the 2012 MDA (hereafter referred to as the follow-up survey). Both surveys employed similar methods of data collection.

### Study setting

The surveys were carried out in the 12 primary schools of Walukuba division, Jinja district of south-eastern Uganda, where schistosomiasis is highly endemic. This setting has been described elsewhere [11].

### Implementation of MDA in the primary schools during the 2011 MDA

During the 2011 MDA, praziquantel was distributed to children on a grade basis, whereby, each grade (year of study) was invited at a time, to receive treatment that was distributed from one pre-arranged central place in each school. The teachers distributed the drug to the children using a standard dose pole and recorded the treatment in the registers. The fear of treatment, lack of teacher support to distribute treatment, and lack of knowledge about schistosomiasis transmission and prevention were some of the major reasons for the low uptake of praziquantel during the 2011 MDA [11]. Lack of funds to conduct trainings, cover teachers’ allowances and to facilitate support supervision were deemed responsible for this state of affairs.

### Strategies to improve uptake of praziquantel in 2012 MDA

Prior to the MDA in 2012, all teachers responsible for distributing and recording treatment in the respective schools received a one days’ refresher training in drug distribution and recording and a small allowance of 2 US $ per teacher. In addition, all the school teachers received T-shirts with inscriptions “NTD control program” on the front and “Get rid of neglected diseases for better health” at the back. Based on the assumption that there are about 12 teachers in each primary school, a total of 12 T-shirts were distributed to each school. In addition, all members of the district health team and the health inspectorate staff also received T-shirts. Furthermore, supervision of MDA by the district health staff was strengthened through provision of transport allowances to facilitate the staff to visit the schools and provide technical support to the teachers during drug distribution. This facilitation was provided by the vector control division, Ministry of Health with support from USAID. It was anticipated that the re-training, strengthened supervision and provision of such incentives to the teachers would uplift their enthusiasm to distribute the drugs to the children and increase uptake.

### Sample size

The uptake of praziquantel used to estimate the sample size for this study was derived from the baseline study [11]. It was assumed that after increasing teacher motivation and strengthening supervision, the uptake would increase by 10%. At 90% power and 95% confidence interval (CI), the sample size required to detect this increase for cross sectional studies is 483 in the follow-up survey according to STATA 10.0 (TX, USA). This sample size was adjusted by 5% non-response to 507. A design effect of 2 was applied to obtain a minimum sample size of 1,014. In this study, 1,020 children were randomly selected from the 12 primary schools to participate in the follow-up survey.

### Sampling and data collection

The study employed similar sampling and data collection methods as those used in the baseline study. The children were randomly selected from grade (year) 4–6 in the 12 primary schools. The number of children to participate in the study from each school and grade was determined by probability proportional to size of the school and grade population. The grade registers, where the names of the children are arranged in alphabetical order, were used as the sampling frame. Systematic sampling was used to select the number of children from each grade to participate in the face to face interviews using a structured questionnaire and undergo a stool examination for *S. mansoni* infection.

### Measures

Children were questioned about their socio-demographic characteristics, whether they took praziquantel at the last MDA and whether they experienced any problems after swallowing the drug. Uptake of praziquantel was measured through self-report and was defined as having received and swallowed praziquantel tablets at the last MDA. In addition, knowledge of schistosomiasis transmission and prevention was measured on a score scale of 1–3. The children were asked to mention the different ways through which the infection can be acquired and prevented, including taking preventive treatment. Various correct options of the disease transmission and prevention methods were listed on the questionnaire and for each of the correct responses, a score of 1 was awarded. Children who mentioned at least one correct method of transmission and two correct methods of prevention scored an aggregate of 3 and were regarded as having correct knowledge of schistosomiasis transmission and control. The prevalence and intensity of *S. mansoni* infection was determined by examining one stool sample on two consecutive days from each child using the Kato-Katz faecal thick smear technique [17].

### Statistical analysis

Analysis was done at the school level to obtain school-specific uptake of praziquantel, prevalence and intensity of *S. mansoni* infection. Mean and standard deviation (S.D) were used to describe continuous data. Within-school clustering was adjusted for using the cluster robust option and robust standard errors were used. 95% CI was used for all the analyses. Chi-square test was used to compare uptake levels, prevalence of infection, knowledge of schistosomiasis transmission and prevention and occurrence of side effects between the baseline and follow-up surveys. Kruskal Wallis test was used to compare intensity of infection between the two surveys. STATA 10.0 (TX, USA) was used for analysis.

### Quality control

We pre-visited the study primary schools to obtain updated sampling frames. Experienced research assistants conducted the interviews. These were trained in data collection methods. Data was checked for completeness and accuracy before leaving the school premises. The principal investigator closely supervised the research assistants during data collection.

### Ethical considerations

Ethical clearance for the study was obtained from Makerere University College of Health Sciences Higher Degrees, Research and Ethics Committee and the Uganda National Council for Science and Technology. Permission to conduct the study in the schools was obtained from the school management. Informed consent was obtained from parents of the children selected to participate in the study. Assent was obtained from the children. Children identified with *S. mansoni* and/or soil-transmitted helminth (STH) infections were treated with praziquantel and/or albendazole. Confidentiality was maintained by using a coding system.

## Results

### Demographic characteristics

Overall, a total of 2,030 children were interviewed and their stool examined for *S. mansoni* infection: 1,010 during the baseline survey and another 1,020 during the follow-up survey. The mean age was 11.6 years (S.D 1.8) and 11.5 years (S.D 1.7) (p = 0.92) at the baseline and follow-up surveys, respectively. The proportion of females was 55.0% and 53.2% during the baseline and follow-up surveys, respectively (p = 0.55). Table 1 shows that the baseline and follow-up samples were comparable in terms of sex, age, and prevalence and intensity of infection with schistosomiasis.

**Table 1:** Comparison of the included children at baseline and follow-up surveys

**Variable**	**Baseline (N = 1,010)**	**Follow-up (N = 1,020)**	**P value**
**Mean age in years (S.D)**	11.6 (1.8)	11.5 (1.7)	0.92
**Number of female children (%)**	555 (55.0%)	543 (53.2%)	0.55
**Prevalence of *S. mansoni *** **in % (n)**	35.0% (354)	32.6% (332)	0.50
**Intensity of *S. mansoni *** **(epg)**	156.7	133.1	0.38

### Self-reported uptake of praziquantel

A significant increase was observed in the proportion of children who reported to have taken praziquantel from 28.2% (95% CI 25.4%-30.9%) at baseline to 48.9% (95% CI 45.8%-52.0%) at the follow-up survey (p < 0.001). Uptake at baseline and follow-up survey ranged from 11.2% to 69.0% and from 13.3% to 60.9%, respectively (Table 2). Uptake among schools that reported moderate uptake at baseline (≥ 50%) did not increase. In schools 4 and 5, uptake decreased from 69.0% to 39.4% and from 64.5% to 41.4%, respectively. In school 9, uptake did not change. In the rest of the schools with an uptake of less than 50% at baseline, uptake increased in 7 (77.8%) of the schools at follow-up.

**Table 2:** Self-reported uptake of praziquantel at baseline and follow-up surveys

**School (no)**	**Baseline (N = 1,010)**			**Follow-up (N = 1,020)**			**P value**
	**n**	**Uptake**	**% uptake (95% CI)**	**n**	**Uptake**	**% uptake (95% CI)**	
1	70	12	17.1 (2.08–48.4)	75	44	58.7 (43.2–73.7)	0.011
2	61	16	26.2 (7.27–52.3)	70	39	55.7 (39.6–72.2)	0.047
3	75	19	25.3 (9.14–51.2)	70	44	62.9 (47.8–77.6)	0.006
4	55	38	69.0 (51.3–82.4)	71	28	39.4 (21.5–59.4)	0.017
5	31	20	64.5 (40.7–84.6)	73	30	41.4 (22.6–59.4)	0.109
6	104	26	25.0 (11.5–47.8)	100	45	45.0 (29.6–60.0)	0.093
7	107	25	23.4 (9.35–45.1)	100	53	53.0 (38.6–66.7)	0.014
8	107	12	11.2 (0.21–38.4)	92	56	60.9 (46.7–73.5)	0.002
9	82	48	58.5 (43.2–72.4)	71	42	59.2 (43.2–74.4)	0.946
10	104	17	16.3 (3.70–43.4)	101	55	54.5 (40.5–68.0)	0.006
11	110	28	25.5 (10.6–44.9)	98	13	13.3 (1.90–45.4)	0.377
12	104	24	23.1 (9.77–46.7)	99	50	50.5 (35.5–64.5)	0.025
Total	1,010	285	28.2 (22.9–33.6)	1,020	499	48.9 (44.4–53.4)	<0.001

### Prevalence and intensity of *S. mansoni* infection

Prevalence and intensity of *S. mansoni* infection was unchanged between the baseline and follow-up surveys with 35.0% (95% CI: 25.4%-37.9%) and 32.6% (95% CI: 29.6%-35.5%) (p = 0.25) and 156.7 epg (95% CI: 116.9-196.5) and 133.1 epg (95% CI: 99.0-167.2) (p = 0.38), respectively. Substantial variations in prevalence (and intensity) of *S. mansoni* infection across the 12 schools were observed at baseline and follow-up surveys and ranged from 16.3% (10.1 epg) to 96.8% (1,419.1 epg) and from 20.7% (18.3 epg) to 78.9% (778.5 epg), respectively (Tables 3 and 4). Significant decrease in prevalence of infection was observed in schools 4, 5 and 6 between the two surveys. Intensity of infection significantly reduced in school 5 but increased in schools 11 and 12 between the two surveys (Table 4).

**Table 3:** **Prevalence of** ***S. mansoni*** **infection at baseline and follow-up**

**School (no)**	**Baseline (N = 1,010)**		**Follow-up (N = 1,020)**		**P value**
	**n**	**% prevalence (95% CI)**	**n**	**% prevalence (95% CI)**	
1	70	24.3 (14.8–36.0)	75	22.7 (13.7–33.7)	0.82
2	61	19.7 (10.5–31.8)	70	21.4 (12.5–32.9)	0.81
3	75	36.0 (25.2–47.9)	70	30.0 (19.6–32.9)	0.44
4	55	94.5 (84.8–98.9)	71	78.9 (67.5–87.6)	0.01
5	31	96.8 (83.2–99.9)	73	56.2 (44.0–67.8)	<0.001
6	104	55.8 (45.6–65.5)	100	24.0 (16.0–33.5)	<0.001
7	107	35.5 (26.5–45.3)	100	29.0 (20.3–38.9)	0.39
8	107	30.0 (21.4–39.5)	92	20.7 (12.9–30.4)	0.13
9	82	34.2 (24.0–45.4)	71	38.0 (26.8–50.3)	0.63
10	104	19.2 (12.1–28.1)	101	30.7 (21.8–40.7)	0.06
11	110	21.0 (13.7–29.7)	98	30.6 (21.6–40.7)	0.11
12	104	16.3 (9.80–24.8)	99	22.2 (14.4–31.7)	0.29
Total	1,010	35.0 (25.4–37.9)	1,020	32.6 (29.6–35.5)	0.25

**Table 4:** **Intensity of** ***S. mansoni*** **infection at baseline and follow-up**

**School (no)**	**Baseline (N = 1,010)**		**Follow-up (N = 1,020)**		**P value**
	**n**	**GM intensity (95% CI)**	**n**	**GM intensity (95% CI)**	
1	70	42.7 (10.5–74.9)	75	30.9 (-3.8–65.6))	0.62
2	61	22.6 (7.3–37.9)	70	40.8 (0.8–80.8)	0.42
3	75	35.7 (17.7–53.7)	70	18.3 (9.1–27.5)	0.09
4	55	1255.5 (798.3–1712)	71	778.5 (435.4–1103.5)	0.08
5	31	1419.1 (746.2–2092)	73	541.5 (274.4–808.6)	0.004
6	104	119.2 (61.6–176.8)	100	87.8 (27.4–148.2)	0.46
7	107	64.3 (27.5–101.1)	100	53.0 (25.9–80.1)	0.63
8	107	55.3 (22.7–87.9)	92	22.0 (9.8–34.2)	0.08
9	82	53.1 (7.5–98.7)	71	111.6 (-16.8 –239.9)	0.37
10	104	58.9 (-15.2–133.0)	101	36.1 (19.7–52.5)	0.56
11	110	11.7 (5.9–17.6)	98	34.6 (18.4–50.8)	0.01
12	104	10.1 (3.8–16.2)	99	35.0 (11.9–58.0)	0.03
Total	1010	156.7 (116.9–196.5)	1,020	133.1 (99.0–167.2)	0.38

GM (Geometric mean) intensity is among the positive cases only.

### Occurrence of side effects and knowledge of schistosomiasis transmission and control

Comparison of other parameters at baseline and follow-up included the occurrence of side effects attributable to praziquantel and knowledge of schistosomiasis transmission and prevention. There was no change in the proportion of children who reported to have experienced side effects after swallowing the drugs at follow-up (46.6%, 95% CI: 42.1%-51.2%) from the baseline (49.8%, 95% CI: 43.8%-55.8%) (p = 0.55). Similarly, there was no change in the proportion of children who had correct knowledge of schistosomiasis transmission and control at follow-up (44.1%, 95% CI: 41.0%-47.2%) from the baseline (45.9%, 95% CI: 42.7%-48.9%) (p = 0.42).

## Discussion

The self-reported uptake of praziquantel among school-age children increased from 28.2% to 48.9% between the two surveys. There was no change in prevalence and intensity of *S. mansoni* infection as well as in levels of knowledge on schistosomiasis transmission and control and occurrence of side effects attributable to uptake of praziquantel.

Although the increase in uptake of praziquantel was statistically significant, it is unlikely to have public health impact as observed by the persistent high prevalence and intensity of infection with schistosomiasis in the schools. The observed increase in self-reported uptake may be attributable to the high level of engagement between the district health team and the teachers during MDA as well as the provision of incentives including facilitation allowances and T-shirts to the school teachers prior to MDA. Such incentives have been found useful in motivating drug distributors [18]. In addition, improvement in self-reported uptake could have been as a result of a more intensive follow-up by the district health team and the presence of researchers in the schools during the pre-visits and drug distribution. Such measured improvements in an aspect of behaviour due to the subjects’ awareness that they are being studied, the so-called “Hawthorne effect”, have been reported in other studies [19,20]. It is worrying that schools with moderate uptake at baseline did not improve and even declined at follow-up. A possible explanation is that concerted efforts to improve uptake, such as the supervision of teachers by the district health team during MDA, were concentrated on schools with very low uptake at baseline. This observation suggests that modification of the conventional school-based MDA programs may possibly improve drug uptake and further studies are needed to test this hypothesis.

The efficacy of praziquantel against *S. mansoni* infection is indisputable [3,8,21-23]. With improved uptake, one would expect a reduction in prevalence and intensity of the infection. Indeed, significant reductions in prevalence were observed in schools (4, 5 and 6) with high uptake levels. It is important to note that this is not a cohort study which follows the same children from baseline to follow-up. In the present study, the two study populations in the two different time-points are not completely identical. The persistent high infections despite the increased uptake of praziquantel could be attributable to the fact that the realized level of uptake is lower than is recommended by the WHO and apparently too low to affect the prevalence and intensity of schistosomiasis among the children. Another factor that could have contributed to the sustained high prevalence and intensities of infection despite the increase in uptake level is that praziquantel is not effective against juvenile schistosomes [24]. The observed eggs may have been a result of immature schistosomes developing into egg laying adult worms during the follow-up period. In this study, 67% of the schools are located within a 5 km radius from lake victoria. Children from schools located closer to the lake frequently visit the lake to fetch water, bathe, to wash, and to swim [25] and therefore get infected with schistosomiasis when they get into contact with contaminated water. This explains the variations in prevalence and geometric mean intensity across the different schools.

The fear of side effects of praziquantel, lack of knowledge about schistosomiasis transmission and prevention and lack of teacher support to take preventive treatment were highlighted as some of the major factors associated with the low uptake among school-age children as reported in the ealier paper from this study [11]. In the follow-up of this study, approximately 50% of the children who received treatment reported to have developed side effects and as such, resistance to swallow the treatment is perhaps inevitable as majority indicated that they would not swallow the drug during the subsequent MDA. Praziquantel causes transient side-effects of the gastro-intestinal and central nervous system including abdominal pain, nausea, vomiting, diarrhea, headache and dizziness, especially when the drug is taken on an empty stomach [23,26,27]. To mitigate the side-effects, the drug should be taken with food [26]. However, most children in rural areas do not take any meals while at school [28] and some take treatment on empty stomachs and experience the side-effects [2,22,29]. Considerable reduction in occurrence of side-effects has been reported in countries where praziquantel was administered with food [2,10]. Therefore, implementing measures for mitigating the side effects attributable to praziquantel, such as providing food during MDA, may improve uptake of the drug among school children. High coverage of praziquantel treatment among school children was achieved by the national control program in Sierra Leone, where a special feeding program for the children to mitigate the side effects was provided [10].

Knowledge of schistosomiasis transmission and control was evidently lacking in more than 50% of the children interviewed, a manifestation of the insufficient health education provided prior to and during MDA. During MDA, distribution of praziquantel takes precedence with diminutive, if any, health education on the infection transmission and control. In the absence of this knowledge and with lack of a clear understanding of the rationale of preventive treatment, there is a risk that the targeted populations may resist the treatment [16,30]. Improved compliance to treatment for schistosomiasis has been reported among school children with adequate knowledge of schistosomiasis transmission and prevention [11,30-32]. In this study, there was a considerable variation in levels of knowledge of schistosomiasis transmission and prevention across the primary schools that ranged from 12.8% to 73.2% at baseline and from 27% to 69% at follow-up. This variation in levels of knowledge of schistosomiasis transmission and prevention could explain the differences in uptake levels of treatment, and prevalence and intensity of infection across the primary schools in the two different time-points. Health education programs for providing school children with information about transmission and prevention are required if sustained drop in prevalence and intensity of *S. mansoni* infection among school children is to be achieved. It is urged that with chemotherapy, prevalence and intensity of decrease when health education is concurrently implemented [30,33].

### Study limitations

The lack of a comparison group makes it difficult to attribute the observed increase in uptake to strengthened supervision and teacher motivation strategy. Moreover, because data on self-reported uptake were derived from interviews, it is possible that the children could have provided desirable answers. However, the comparison of our findings with those of previous studies in other settings lend credibility to our observations and suggest that the observed increase in uptake could have ensued from the strengthened supervision by the district health team and teacher motivation to distribute the drugs [32,33]. The second limitation is the relatively low intrinsic capacity of the Kato-Katz technique to recover parasite eggs from specimens of low infection intensities compared to other parasitological methods [34,35]. To increase its sensitivity during the study, two samples were taken from each child and two slides were prepared per fecal sample.

## Conclusions

Strengthening supervision and increasing teacher motivation to distribute treatment is an important but not sufficient strategy for improving uptake of praziquantel. Uptake must be further increased to significantly reduce levels of transmission of infection. The strategies for improving uptake could be health education and/or measures to mitigate the side effects of the drug, such as provision of a snack during MDA. Randomized controlled trial studies should be undertaken to evaluate such interventions.

## Competing interests

The authors declare that they have no competing interests.

## Authors’ contributions

SM, AK, FN and AO participated in the design of the study. SM participated in data collection. SM and FN performed the statistical analysis. SM drafted the article. All authors reviewed the manuscript. All authors read and approved the final manuscript.

## Pre-publication history

The pre-publication history for this paper can be accessed here:

http://www.biomedcentral.com/1471-2334/13/590/prepub
